# Membrane vesicles from antibiotic‐resistant *Staphylococcus aureus* transfer antibiotic‐resistance to antibiotic‐susceptible *Escherichia coli*

**DOI:** 10.1111/jam.15449

**Published:** 2022-02-11

**Authors:** Ae Rin Lee, Seong Bin Park, Si Won Kim, Jae Wook Jung, Jin Hong Chun, Jaesung Kim, Young Rim Kim, Jassy Mary S. Lazarte, Ho Bin Jang, Kim D. Thompson, Myunghwan Jung, Min Woo Ha, Tae Sung Jung

**Affiliations:** ^1^ Laboratory of Aquatic Animal Diseases, Research Institute of Natural Science, College of Veterinary Medicine Gyeongsang National University Jinju‐si Gyeongsangnam‐do Republic of Korea; ^2^ Coastal Research Extension Center Mississippi State University Mississippi State Mississippi USA; ^3^ Department of Microbiology, Institute for Viral Diseases College of Medicine, Korea University Seoul Republic of Korea; ^4^ Moredun Research Institute Pentlands Science Park Midlothian UK; ^5^ Department of Microbiology College of Medicine, Gyeongsang National University Jinju Republic of Korea; ^6^ Department of Convergence Medical Science College of Medicine, Gyeongsang National University Jinju Republic of Korea; ^7^ College of Pharmacy Jeju National University Jeju Republic of Korea; ^8^ Centre for Marine Bioproducts Development Flinders University Bedford Park South Australia Australia

**Keywords:** antibiotic‐resistant bacteria, antibiotic‐susceptible bacteria, gram‐negative bacteria, gram‐positive bacteria, membrane vesicles (MVs), vesicle‐mediated transferring of antimicrobial resistance

## Abstract

**Aim:**

Bacteria naturally produce membrane vesicles (MVs), which have been shown to contribute to the spread of multi‐drug resistant bacteria (MDR) by delivering antibiotic‐resistant substances to antibiotic‐susceptible bacteria. Here, we aim to show that MVs from Gram‐positive bacteria are capable of transferring β‐lactam antibiotic‐resistant substances to antibiotic‐sensitive Gram‐negative bacteria.

**Materials and Methods:**

MVs were collected from a methicillin‐resistant strain of *Staphylococcus aureus* (MRSA) and vesicle‐mediated fusion with antimicrobial‐sensitive *Escherichia coli* (RC85). It was performed by exposing the bacteria to the MVs to develop antimicrobial‐resistant *E. coli* (RC85‐T).

**Results:**

The RC85‐T exhibited a higher resistance to β‐lactam antibiotics compared to the parent strain. Although the secretion rates of the MVs from RC85‐T and the parent strain were nearly equal, the β‐lactamase activity of the MVs from RC85‐T was 12‐times higher than that of MVs from the parent strain, based on equivalent protein concentrations. Moreover, MVs secreted by RC85‐T were able to protect β‐lactam‐susceptible *E. coli* from β‐lactam antibiotic‐induced growth inhibition in a dose‐dependent manner.

**Conclusion:**

MVs play a role in transferring substances from Gram‐positive to Gram‐negative bacteria, shown by the release of MVs from RC85‐T that were able to protect β‐lactam‐susceptible bacteria from β‐lactam antibiotics.

**Significance and impact of study:**

MVs are involved in the emergence of antibiotic‐resistant strains in a mixed bacterial culture, helping us to understand how the spread of multidrug‐resistant bacteria could be reduced.

## INTRODUCTION

Since the discovery of penicillin, antibiotics have helped prolong the lifespan of humans (Shin, 2017). However, the abuse and misuse of antibiotics have resulted in the emergence of antibiotic‐resistant bacteria, known as superbugs or multidrug‐resistant (MDR) bacteria (Nazir et al., 2012; Singh, 2018). These MDR bacteria have prompted the development of improved antibiotics, but new strains of bacteria have subsequently emerged that are able to resist these new and improved antibiotics. The widespread increase in MDR bacteria is a major concern for the medical community. In fact, it is predicted that by 2050, the harm caused by multi‐drug resistant bacteria will exceed that of cancer, in terms of mortality and economic losses worldwide (Center of Disease Control and Prevention, 2013; O'Neill, 2016). Thus, it is imperative to understand the mechanisms behind the ability of bacteria to counteract the effects of antibiotics to prevent the further spread of MDR bacteria.

Different mechanisms are involved in protecting bacteria from the action of antibiotics (Poole, 2002). Some bacteria use an efflux pump, which removes antibiotics from within the bacteria to the outside (Chopra & Roberts, 2001; Masuda et al., 1999; Poole, 2000), some block the permeability of antibiotics (Hancock, 1997; Nikaido, 1994), others change the site of action so it is no longer affected by the antibiotic (Leclercq & Courvalin, 1991; Weisblum, 1995), while others express enzymes, which can inactivate specific antibiotics (Livermore, 1995; Matsuoka et al., 1998; Thomson & Moland, 2000). Some antibiotic‐sensitive bacteria form stronger biofilms or turn into persisters in the presence of antibiotics. Furthermore, transformation, transduction, conjugation, and vesiduction are key mechanisms leading to genetic variation within the bacteria, which can result in improved bacterial survival against antibiotics, as well as their ability to directly transfer antibiotic‐resistant genes to other bacteria (Soler & Forterre, 2020; Von Wintersdorff et al., 2016). These mechanisms of horizontal transfer of genes together with the heritably of different mutational events may give rise to potential phenotypes with genetically stable resistance via natural selection. On the other hand, studies have demonstrated that membrane vesicles (MVs) secreted from antibiotic‐resistant bacteria can contribute to antibiotic resistance within a heterogeneous bacterial community in a non‐heritable manner (Kim et al., 2018).

Regardless of the bacterial species, the MVs secreted extracellularly have been referred to as extracellular vesicles (Gill et al., 2019; Kim, Jang, et al., 2015; Kim, Lee, et al., 2015; Kim et al., 2018; Kulkarni et al., 2015; Turner et al., 2016). Some bacteria also produce intracellular vesicles. There are two kinds of MVs secreted by Gram‐negative bacteria, outer membrane vesicles (OMVs) and inner‐outer membrane vesicles (I‐OMVs). Since the Gram‐positive bacterial cell has only one membrane, the Gram‐positive bacteria produce only one type of vesicles—the MVs. In general, most Gram‐negative MVs form from the outer part of bacteria, wrapping around substances that make up the surrounding periplasm, and are released from the cell to form nano‐sized spherical vesicles (Collins, 2011; Kuehn & Kesty, 2005; Mashburn‐Warren et al., 2008). In the process of their formation, the MVs can incorporate proteins, glycoproteins, lipids, and genetic material (Quan et al., 2018; Rumbo et al., 2011; Yaron et al., 2000). Vesicles secreted from bacteria can transfer their contents to other bacterial cells or host cells located nearby through adhesion and endocytosis (Kadurugamuwa & Beveridge, 1999; Meyer & Fives‐Taylor, 1993). Gram‐negative MVs have a stable structure surrounded by a bi‐layered membrane, and these allow the bacterium to interact with the outside without direct movement (Fulsundar et al., 2014; Lee et al., 2013). However, a few studies have demonstrated the biogenesis and composition of MVs secreted from Gram‐positive bacteria, showing them to lack an outer membrane layer, but are surrounded by thick layers of peptidoglycan (Brown et al., 2015; Liu et al., 2018; Nagakubo et al., 2020; Toyofuku et al., 2019). Enzymes, such as endolysin and autolysin may play a crucial role in MVs formation by making holes in the peptidoglycan layers, as shown with *Bacillus subtilis* or damage to the cell wall of *Staphylococcus aureus* (Andreoni et al., 2019; Toyofuku et al., 2019). Although studies have reported that the biochemical and physical characteristics of MVs are different from that of the cell membrane, it is largely unknown how MVs form from the thick peptidoglycan layers of Gram‐positive bacteria (Brown et al., 2015; Liu et al., 2018).

The aim of the present study was to demonstrate that antibiotic resistance can be transferred to antibiotic‐sensitive Gram‐negative bacteria by MVs from Gram‐positive antibiotic‐resistant bacteria.

## MATERIALS AND METHODS

### Bacterial strains

The methicillin‐resistant *S. aureus* strain t324‐ST541‐V methicillin‐resistant strain of *S. aureus* (MRSA; ST541) was isolated from a pig sample obtained from the Animal and Plant Quarantine Agency Korea (Moon et al., 2016), while the β‐lactam‐sensitive *S. aureus* strain ATCC29213 was purchased from American Type Cell Collection (ATCC). The bacteria were grown at 37°C in either Luria‐Bertani (LB; BD) broth or tryptone soya agar (BD) on an orbital shaker. Antimicrobial‐sensitive *Escherichia coli* RC85 (Kim et al., 2018) was used as the target bacterium. RC85 and a vesicle‐mediated antibiotic‐resistance transferred strain (RC85‐T) were grown in either LB broth or on LB agar, again at 37°C with orbital shaking. Growth was monitored by measuring absorbance at 600 nm (OD600) using an SPL (SPL life science) microplate spectrophotometer (Bio‐Rad).

### Isolation and visualization of MVs

Purification of MVs from ST541, RC85, and RC85‐T cells was performed as previously described (Kim, Seo, et al., 2020). Briefly, the bacterial culture was centrifuged at 6000× g for 20 min, and the supernatant was filtered through 0.45‐μm pore‐sized vacuum filters. The filtered supernatant was concentrated by ultrafiltration using a QuixStand Benchtop system (GE Healthcare). This was then centrifuged at 150,000 × g at 4°C for 3 h, and the MVs were purified through continuous sucrose density gradient at 120,000× g at 4°C for 18 h. The MV band was selected and washed with 20 ml of 10 mM Tris–HCl (pH 8.0). These pure MVs were then centrifuged for 3 h at 150,000× g at 4°C. The final pellet was washed and resuspended in the same buffer and filtered through 0.2‐μm filter. The resulting MVs suspensions were streaked onto LB agar to confirm that the MVs preparations were free of bacteria and were subsequently stored at −80°C until used. The protein yield of the vesicle samples from ST541, RC85‐T, and RC85 were measured using a BCA protein assay kit according to the manufacturer's manual (ThermoFisher Scientific).

Transmission electron microscopy (TEM) of vesicles from ST541, RC85‐T, and RC85 cells were performed as previously described (Kim et al., 2018; Kim, Seo, et al., 2020). Vesicles were placed on a carbon‐coated grid (Ted Pella) that had been glow‐discharged for 3 min in air. The grid was immediately dyed with 1% (w/v) uranyl acetate and then examined using a Tecnai G2 Spirit Twin TEM system (FEI) under an acceleration voltage of 120 kV.

### Transferring MVs‐mediated antibiotic‐resistance

The transfer of antibiotic‐resistance using MVs was performed according to a previously described method with minor modifications (Rumbo et al., 2011; Yaron et al., 2000). Briefly, antimicrobial‐sensitive *E. coli* (RC85) was cultured in LB broth at 37°C to an optical density of 1.0 at 600 nm (OD 600). Cells were diluted with phosphate buffered saline (PBS) at 5 × 10^5^ CFU/ml. The diluted cells (100 μl) were mixed with purified MVs containing 100 μg of protein and added to 800 μl of cold super optimal broth medium (2% tryptone, 0.5% yeast extract, 0.4% glucose, 10 mM NaCl, 2.5 mM KCl, 5 mM MgCl_2_, 5 mM MgSO_4_). This mixture was incubated statically for 4 h at 37°C. Ten millilitres of LB broth was then added to the mixture, and the incubation continued for 12 h at 37°C with shaking (150 rpm). The broth culture was grown for a further 12 h, then diluted to 1% (v/v) using PBS and smeared on 50 μg/ml of ampicillin agar. After culture, several colonies were randomly picked and passaged up to five times on the same concentration of ampicillin agar. One colony was randomly picked and named RC85‐T. The negative control was prepared with identical conditions, but PBS was used in place of 100 μg of MVs. All experiments were performed in duplicate and repeated twice. To ensure MVs were free of DNA which may induce the transformation, the MVs samples were treated with 2 U of DNase I (Invitrogen Life Technologies) for 2 h according to the manufacturer's instructions. The DNase I was inactivated by the addition of 25 mM of EDTA. Colonies from RC85‐T were identified by matrix‐assisted laser desorption ionization‐time of flight mass spectrometry (MALDI Biotyper; Bruker Daltonik; Kim, Jang, et al., 2015; Kim, Lee, et al., 2015) to confirm that they were *E. coli*.

### Particle size distribution and measurement of zeta potential of MVs

Dynamic light scattering (DLS) of vesicles obtained from ST541, RC85‐T, and RC85 cells were performed as described previously (Kim et al., 2018; Kim, Seo, et al., 2020). Each set of vesicles were characterized by measuring their particle size distribution and zeta potential. The diameter of the collected vesicles was measured at 25°C by DLS using a Nano ZS instrument (Malvern Instruments) and the Zetasizer software (version 7.11; Malvern Instruments). Three independent measurements (15 experimental runs for each measurement) were averaged to obtain an average intensity‐weighted diameter. The zeta potential was measured using a Nano ZS instrument (Malvern) with a Helium‐Neon laser (633 nm) at 25°C.

### Determination of minimum inhibitory concentrations

The minimum inhibitory concentration (MIC) was evaluated for ST541, ATCC29213, RC85, and RC85‐T strains. To confirm the role of MVs in interrupting antibiotic activity, several antimicrobials namely, ampicillin, cefotaxime, cefoperazone, colistin, gentamicin, and tetracycline were utilized. Eight β‐lactam antibiotics, i.e., ampicillin, amoxicillin, cefalexin, cefazolin, cefoperazone, cefotaxime, cloxacillin, and methicillin (Sigma‐Aldrich), and five other classes of antibiotics, i.e. colistin, gentamicin, nalidixic acid, streptomycin, and tetracycline (Sigma‐Aldrich) were selected to compare relative resistance levels of RC85‐T with that of RC85. The MIC was determined by broth‐dilution method in 96‐well plates (Andrews, 2001; Kim et al., 2018). according to Clinical and Laboratory Standards Institute guidelines, except that cation‐adjusted Muller Hinton broth was substituted with LB. The MIC values were measured from three independent experiments.

### Effect of MVs on the growth of bacteria in the presence of β‐lactam antibiotic

To evaluate the effect of MVs on the growth of bacteria in the presence of a β‐lactam antibiotic, a previously described experiment was performed with slight modifications (Kim et al., 2018; Kim, Lee, et al., 2020). The β‐lactam antibiotic used was from the penicillin family (ampicillin). Ampicillin was used at concentrations known to inhibit RC85 growth (32 μg/ml). Cultured RC85 cells (5 × 10^5^ CFU/ml) were inoculated into medium containing the antibiotic and 30 μg/ml of MVs from RC85‐T and RC85 cells. RC85 in the antibiotic‐free medium was used as a positive control, while the negative control consisted of bacteria and growth‐inhibitory concentrations of the antibiotic. All tubes were incubated at 37°C with shaking at 150 rpm. All experiments were performed in the dark to exclude the effect of light on the stability of the antibiotic used. The bacterial growth curves at OD600 were recorded at 3‐h intervals up to 36 h. Experiments were performed using three independent sets of bacterial cultures.

### Quantification of β‐lactamase activity

To test the differences in β‐lactamase activity of MVs and whole cell lysates (WCL) between RC85‐T and RC85 cells, a colorimetric β‐lactamase activity assay kit (BioVision) was used following the manufacturer's instructions. The assay is based on the hydrolysis of nitrocefin, a chromogenic cephalosporin producing a coloured product that can be measured spectrophotometrically (OD490). The quantity of enzyme capable of hydrolysing 1.0 μM of nitrocefin/min at 25°C corresponds to 1 U of β‐lactamase. Equal concentrations of each sample (1.7 μg) were dispensed into the wells of a clear flat‐bottomed 96‐well, and nitrocefin and buffer (provided in the kit) were added to make a final volume of 100 μl. The absorbance at OD490 was immediately measured in kinetic mode for 60 min at 25°C. For all measurements, three independent experiments were performed. A standard curve was generated using 0, 4, 8, 12, 16, and 20 nM of nitrocefin, and the specific β‐lactamase activity of each sample was expressed in milliunits/milligram of protein.

### Measurement of antibiotic concentrations

Measurement of β‐lactam antibiotic concentrations was carried out as previously described (Kim et al., 2018; Kim, Seo, et al., 2020) with slight modifications. The effect of MVs from RC85‐T and RC85 on the degradation of ampicillin in a cell‐free system was analysed through liquid chromatography/electrospray ionization mass spectrometry (LC‐ESI‐QQQ‐MS/MS; 6420 Triple Quad LC/MS; Agilent). Thirty μg/ml of each respective MV sample in PBS was mixed with ampicillin (20 μg/ml). Filtered PBS containing respective antibiotics without MVs was used as a positive control. All samples were incubated at 37°C with shaking at 150 rpm and diluted 20‐fold prior to analysis. The concentrations of antibiotic were recorded at specific time points (ampicillin; 3 h) in triplicate. For the quantification of antibiotic, at least two or more transitions were selected for each analyte and the positive electric spray ionization (ESI+) was used with multiple reaction monitoring mode. The MassHunter software (version B.06.00; Agilent) was used to process the LC–MS/MS data and quantification of the analytes.

### Preparation of WCL

WCL were purified as described previously with some modifications (Kim et al., 2018; Kim, Seo, et al., 2020). The WCLs of RC85‐T and RC85 were obtained from cells grown in LB for 4 h 30 min at 37°C, pelleted at 5000× g for 30 min, and washed with 1× phosphate buffered saline (PBS, pH 7.0). The pelleted cells were suspended to 4 ml/g cells in chilled lysis buffer (50 mM Tris–HCl at pH 7.5, 100 mM NaCl, 5 mM dithiothreitol (DTT), and 1 mM phenylmethylsulfonyl fluoride). After incubation on ice for 10 min, cell suspensions were sonicated on ice using ten cycles of 10 s bursts with 30 s cooling intervals. The resulting crude extracts were centrifuged at 12,000× g for 5 min at 4°C to remove cell debris. The WCL pellet was resuspended in 10 mM Tris–HCl (pH 8.0). The protein concentrations of WCL were quantified using BCA protein assay kit according to the manufacturer's instructions (Bio‐Rad protein assay kit).

### Sodium dodecyl sulfate‐polyacrylamide gel electrophoresis

Sodium dodecyl sulfate‐polyacrylamide gel electrophoresis (SDS‐PAGE) was carried out according to Laemmli’s method (Laemmli, 1970). Equal amounts of WCL and MVs samples from RC85‐T and RC85 cells were mixed with 1× sample buffer and then separated by SDS‐PAGE using a 10% (w/v) acrylamide separating gel. The gel was then stained with Bio‐safe Coomassie G‐250 (Bio‐Rad) and destained with destaining solution (deionized water with 10% Acetic acid and 20% Methanol).

### In‐gel digestion

The SDS‐PAGE lane containing all protein bands was excised from top to bottom using a razor blade, excised gel slices were washed twice with 100 μl of distilled water for 15 min at RT. They were destained with 50% acetonitrile and shrunk with 100% acetonitrile. Proteins in the gel were then soaked in 500 μl of 5 mM ammonium bicarbonate for 5 min at RT. The ammonium bicarbonate solution was removed by pipetting and 500 μl acetonitrile was then added and incubated for 5 min at RT. After acetonitrile incubation, the slices were dried in a vacuum, and were incubated again in 10 mM DTT/0.1 M ammonium bicarbonate for 45 min at 56°C, and then alkylated by incubation with 55 mM iodoacetamide/0.1 M ammonium bicarbonate for 30 min at room temperature in the dark. After alkylation, the slices were dried again then rehydrated in 5 μl of digestion buffer that contained 25 mM ammonium bicarbonate, 0.1% *n*‐octyl glucoside, and 50 ng/ml of sequencing grade trypsin (Promega). After rehydration, the band slices were incubated overnight at 37°C in 50 μl digestion buffer (without trypsin) to allow enzymatic cleavage in siliconized tubes. Approximately 0.1 μg of protease was used for one gel band. Peptides were extracted from the gel slices with 66% acetonitrile, 33% water, 0.1% trifluoroacetic acid (TFA). After centrifugation, the peptides were transferred into a new tube and dried with a Speedvac system (Hanil). The dried peptides from the gel slices were stored at −80°C before analysis.

### LC–MS/MS analysis

LC–MS/MS analysis was carried out as previously described (Khang et al., 2014; Park et al., 2011), with some modifications. Each peptide mixture was resuspended in 0.1% TFA and injected onto an analytical column (Zorbax 300SB‐C18 75 μm i.d. × 15 cm column; Agilent) via a trap column (Zorbax 300SB‐C18 300 μm i.d. × 5 mm column; Agilent). The peptides were separated in an acetonitrile gradient of buffer A (0.1% formic acid in water) and buffer B (0.1% formic acid in pure acetonitrile) at a constant flow rate of 0.2 μL/min, using an Agilent 100 series nano HPLC system coupled on‐line to an LTQ ion‐trap mass spectrometer (Thermo Fisher Scientific). A full‐scan mode (m/z 350–1600) was enabled, and each survey MS scan was followed by three MS/MS scans. The utilized ESI‐Q‐TOF ion source parameters were as follows: ion spray voltage, 2.2 kV; capillary voltage, 24 V; and capillary temperature, 200°C. The rolling collision energy was set to 35%.

### Quantitative protein profiling, statistics, database searching and in silico analysis of associated proteins

The LC/MS data were analysed with DeCyder MS software (version 2.0; GE Healthcare). The MS/MS spectra of the peptide peaks were searched against the SwissProt bacterial database (*E. coli*) using MascotTM 2.3 (Matrix Science). Detailed information about the methods of quantitative protein profiling, statistics and database searching can be found in the Supporting Information. Software Tool for Researching Annotations of Proteins version 1.5 (Boston University School of Medicine, USA), which was used to classify the differentially expressed proteins by their gene ontology terms, such as biological process, cellular component, and molecular function. We plan to publish the complete proteomic data elsewhere.

### Statistical analysis and data availability

Statistical analysis was performed using Graphpad Prism, version 8.1.1. (GraphPad). Significant differences were determined by ANOVA. Data are presented as mean ± standard deviation (SD). The difference was considered statistically significant at *p* < 0.05.

## RESULTS

### Physical characterization of MRSA MVs

**FIGURE 1:**
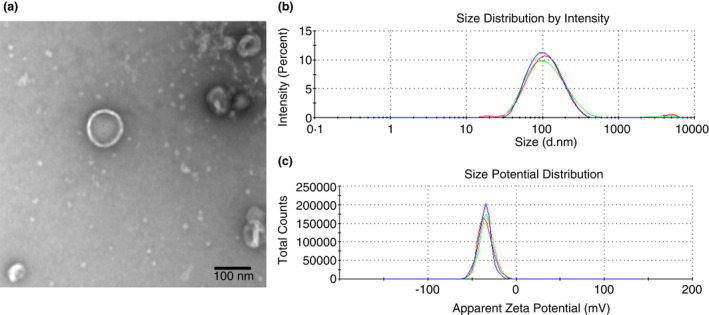
Physical characterizations of membrane vesicles (MVs) derived from methicillin‐resistant *Staphylococcus aureus* (MRSA) ST541 cells. Transmission electron microscopy image of MVs derived from ST541 cells (a) (scale bar: 100 nm). The size distribution MVs released from ST541 cells (b), as assessed by dynamic light scattering. Three independent analyses were performed. (c) The zeta potential of MVs from ST541 cells was measured by the zeta‐sizer and each experiment was performed in triplicate

The MVs from MRSA ST541 cells were isolated and examined using TEM and DLS. The TEM image of MRSA ST541 MVs revealed bi‐layered spherical structures (Figure 1), while DLS indicated the average diameter of ST541 MVs as 97.18 ± 0.68 nm (Figure 1 and Table 1). Their polydispersity index (PDI) factor was measured as 0.226 ± 0.007, signifying that the MVs were monodispersed. Their zeta potentials were −35.77 ± 0.83 mV, which suggests that the particles were in a stable state (Figure 1; Table 1).

**TABLE 1:** Physical characterization of membrane vesicles (MVs) from *Staphylococcus aureus* ST541, *Escherichia coli* RC85‐T and *E. coli* RC85 cells

Name	Size (nm)	Polydispersity (d nm)	*Z*‐potential (mV)
ST541 MVs	97.18 ± 0.68	0.226 ± 0.006	35.77 ± 0.83
RC85‐T MVs	81.18 ± 0.49	0.269 ± 0.007	−23.60 ± 1.24
RC85 MVs	66.90 ± 0.71	0.266 ± 0.006	−26.77 ± 1.68

### MVs protect β‐lactam‐susceptible *S. aureus* cells against β‐lactam antibiotics

**FIGURE 2:**
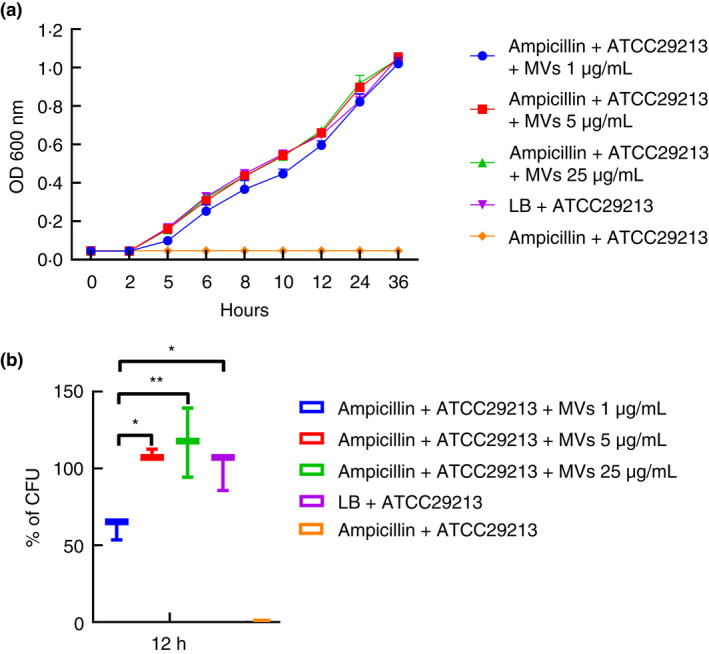
Membrane vesicles (MVs) from MRSA ST541 can protect β‐lactam‐susceptible *Staphylococcus aureus*, preventing β‐lactam antibiotic‐induced growth inhibition in the bacteria. (a) Representative growth profiles of β‐lactam‐susceptible *S. aureus* cells in the presence of growth‐inhibiting concentrations of β‐lactam antibiotics. The growth‐inhibiting concentrations of antibiotics were ampicillin, 60 μg/ml. The data were presented as means and standard error of the mean (SEMs) of at least three independent experiments. (b) The survival percentages of β‐lactam‐susceptible *S. aureus* cells in the presence of the above‐listed growth‐inhibiting concentrations of antibiotics and MVs were calculated by bacterial counts of cultures at a certain time point (ampicillin, 12 h). CFU of *S. aureus* cells in medium without any antibiotics were used as a positive control and taken as 100%, to which corresponding CFU of samples were compared. The data were presented as means and SEMs of three independent experiments. *p < 0.05, **p < 0.01

The MIC of MRSA strain ST541 and susceptible *S. aureus* ATCC29213 were measured, and the results are shown in Table S1. The growth kinetics presented in Figure 2 showed that ST541 MVs can protect susceptible ATCC29213 bacteria against ampicillin at a higher concentration than the MIC of ATCC29213. To determine the extent of the MV‐mediated protection of ATCC29213 cells against ampicillin, we carried out quantitative plate assays based on growth kinetics. Results showed that MVs can protect ATCC29213, allowing it to tolerate antibiotic exposures above the MIC of untreated bacteria (Figure 2).

### MV‐mediated antibiotic‐resistance transferring and characterization of *E. coli* RC85‐T

**FIGURE 3:**
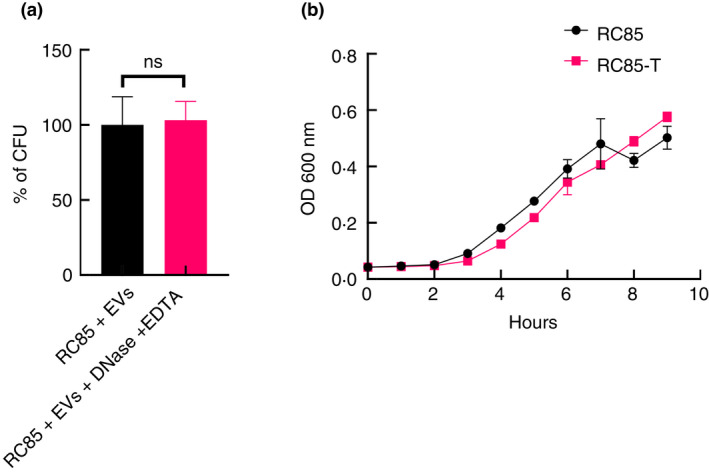
Percentage of antibiotic‐resistance acquired in RC85 after incubating with membrane vesicles (MVs) treated with and without DNase and representative growth profiles of RC85 and RC85‐T. (a) CFU of cells gaining antibiotic‐resistant in DNase‐treated samples, used as a positive control and taken as 100%, with the corresponding CFU of samples compared to this. The data were presented as means and SEMs of three independent experiments. The abbreviation ‘ns’ means not significant. (b) Growth profiles of respective samples, as obtained by measuring absorbance at 600 nm for up to 10 h every 2 h. The data are presented as means and SEMs of three independent experiments

To establish the MV‐mediated transfer of antibiotic‐resistant substances, β‐lactam‐sensitive *E. coli* RC85 was exposed to MVs purified from MRSA ST541 as described in Materials and Methods. The strain obtaining antibiotic‐resistance was designated as RC85‐T. When DNAse‐treated MVs were used to confirm that they were free of DNA, no significant difference was seen in the percentage of bacteria gaining antibiotic resistance, with and without DNase treatment (Figure 3a). Colonies from each selected RC85‐T sample (*n* = 10, by MALDI Biotyper colonies per sample) growing on LB agar plates with ampicillin were randomly selected and identified to check for contamination by other bacteria. The colonies were identified as *E. coli* at a species level, the same as the recipient RC85 strain (Table S2). The RC85‐T strain grew well in LB medium and exhibited a slightly slower but similar logarithmic phase growth compared to RC85 (Figure 3b).

### Alteration in MIC

The MIC of RC85‐T strain was compared with parent strain RC85 (Table 2), using eight kinds of β‐lactam antibiotics, as well as other classes of antibiotics. With ampicillin, amoxicillin, and cefalexin, RC85‐T had higher MIC levels than RC85. However, there was no significant difference in MIC levels with cefazolin, cefoperazone, cefotaxime, cloxacillin, methicillin, and other antibiotics, indicating that RC85‐T had selectively acquired antibiotic resistance for specific β‐lactam antibiotics.

**TABLE 2:** Antibiotic susceptibility profiles of RC85 and RC85‐T strains

Class	Antibiotics	MIC (μg/ml)	
		RC85	RC85‐T
β‐lactams	Ampicillin	16	128
	Amoxicillin	16	128
	Cefalexin	8	64
	Cefazolin	4	8
	Cefoperazone	≤0.5	≤0.5
	Cefotaxime	≤0.5	≤0.5
	Cloxacillin	≥512	≥512
	Methicillin	≥512	≥512
Aminoglycosides	Gentamicin	2	4
	Kanamycin	16	16
	Streptomycin	16	32
Polymyxins	Colistin	32	16
Tetracyclines	Tetracycline	2	1
	Doxycycline	2	2
Quinolone	Nalidixic acid	≥512	≥512

Abbreviation: MIC, minimum inhibitory concentration.

### Physical characterization of MVs secreted from *E. coli* RC85‐T and *E. coli* RC85 cells grown in vitro

**FIGURE 4:**
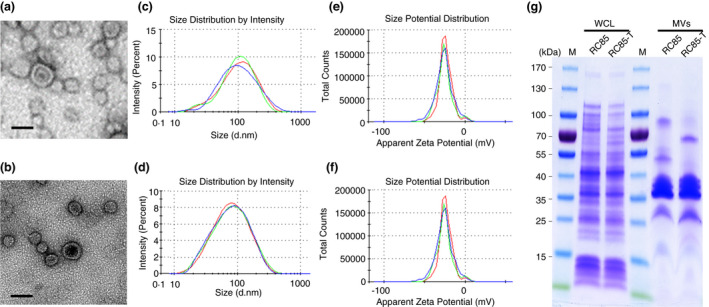
Physical characterizations of membrane vesicles (MVs) from RC85‐T and RC85 cells. Transmission electron microscope images of MVs derived from RC85‐T cells (a), and RC85 cells (b) (scale bar: 100 nm); the size distribution of MVs released by RC85‐T cells (c), and RC85 cells (d), as assessed by the zeta‐sizer (three independent analyses were performed;); the zeta potential of MVs released by RC85‐T cells (e), and RC85 cells (f) were measured by the zeta‐sizer and each experiment was performed in triplicate; equal amount of whole cell lysates (WCLs), MVs from RC85‐T and RC85 (g) cells were separated by 10% SDS‐PAGE and then the protein profiles were visualized using Coomassie staining

The MVs of RC85‐T and RC85 cells were isolated and examined using TEM and DLS. The TEM image of RC85‐T and RC85 MVs revealed bi‐layered spherical structures (Figure 4a,b). DLS determined that the average diameter of RC85‐T MVs (81.18 ± 0.49 nm) was slightly larger than that of RC85 MVs (66.90 ± 0.71 nm; Figure 4c,d; Table 1). Their polydispersity index (PDI) was below 0.3 respectively, indicating that the vesicles were monodispersed (Table 1). Their zeta potential was more negative than −30 mV, which signifies that there was no significant difference in the stability of the particles (Figure 4e,f; Table 1). SDS‐PAGE was performed on purified MVs and WCL of bacteria to investigate whether the proteins of MVs originated from bacteria and to compare changes in the composition of bacteria and MVs (Figure 4g). Comparison of protein profiles between WCLs and MVs fractions showed differences in both protein composition and expression levels.

### Production rates and β‐lactamase activity of MVs

**FIGURE 5:**
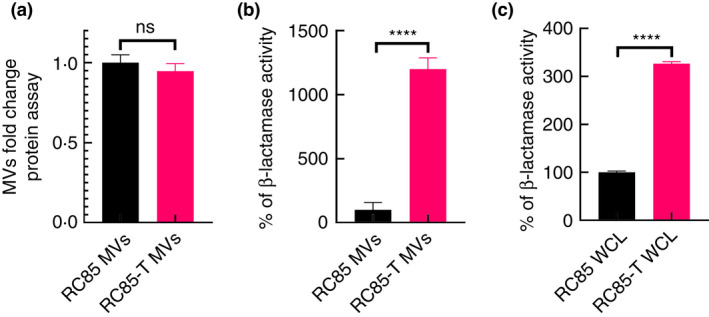
Production of membrane vesicles (MVs) isolated from RC85‐T and RC85 cells and differences in β‐lactamase activity between whole cell lysates (WCLs) and MVs. (a) Protein quantification of MVs was conducted using a BCA protein assay. The protein concentration was averaged and normalized to untreated controls to adjust fold change. Data represent means ± SEMs of three independent experiments. The abbreviation ‘ns’ means not significant. (b) β‐lactamase activity of MVs from RC85‐T cells and RC85 cells. (c) β‐lactamase activity of WCLs from RC85‐T and RC85 cells. Respective samples, as obtained by measuring absorbance at 490 nm. The data are presented as means and SEMs of three independent experiments. ****p < 0.0001

A protein assay was performed to investigate differences in production of MVs between RC85‐T and RC85 cells (Figure 5a). The production of RC85‐T MVs was found to be similar to that of the RC85 MVs. Differences in β‐lactamase activity between WCL and MVs of RC85‐T and RC85 cells over time are shown in Figure 5b,c. The β‐lactamase activity of RC85‐T and RC85 WCLs was 18.6 mU/mg and 5.7 mU/mg respectively, representing a 3.3 times difference in activity between the WCLs. On the other hand, β‐lactamase activity of the MVs was 2.4 mU/mg (RC85‐T) and 0.2 mU/mg (RC85), with the RC85‐T MVs having 12‐times more activity compared to the RC85 MVs.

### MVs from *E. coli* RC85‐T protect *E. coli* RC85 in the presence of β‐lactam antibiotic

**FIGURE 6:**
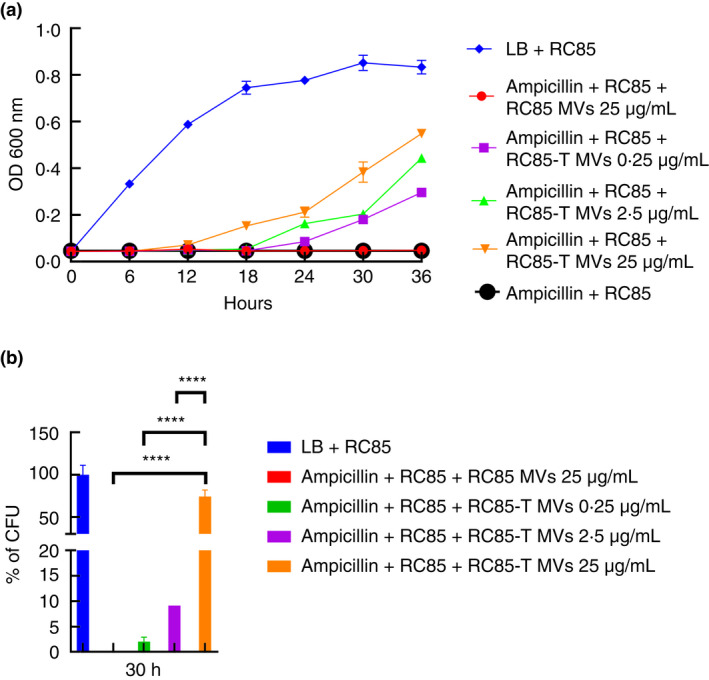
Membrane vesicles (MVs) from RC85‐T cells can protect β‐lactam‐susceptible RC85 from ampicillin‐induced growth inhibition. The growth‐inhibiting concentration of ampicillin was 32 μg/ml. The data represents means and SEMs for at least three independent experiments. (a) the representative growth profiles of β‐lactam‐susceptible RC85 cells in the presence of growth‐inhibiting concentrations of ampicillin and MVs were calculated from bacterial counts of cultures (CFU‐ colony forming units) taken at different time points (ampicillin, 30 h). (b) CFU of RC85 cells in medium without any antibiotics were used as a positive control and taken as 100%, from which the corresponding CFU were determined. The data were presented as means and SEMs of three independent experiments. ****p < 0.0001

**FIGURE 7:**
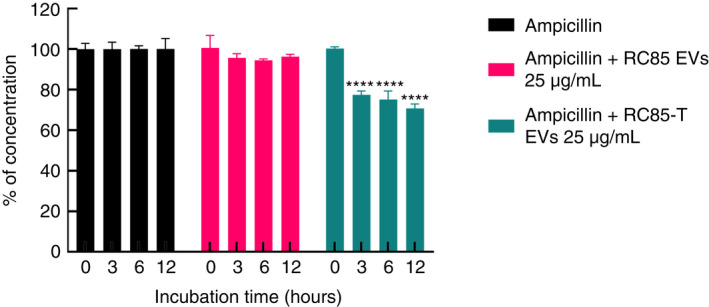
LC‐QQQ‐based assessment of ampicillin concentration following incubation with RC85‐T or RC85 membrane vesicles (MVs) in a cell‐free system. The initial concentration of ampicillin was 20 μg/ml. Twenty‐five micrograms per millilitre of MVs of RC85‐T and RC85 in sterilized 1× PBS were mixed with ampicillin. Filtered 1× PBS containing the ampicillin without MVs were used as a positive control and taken as 100% antibiotic concentration, from which the corresponding concentrations of antibiotic in samples treated with RC85‐T MVs or RC85 MVs were analysed. The concentrations of antibiotics were recorded in triplicate at different time points. Bars indicate standard deviations. **** *p* < 0.0001

To investigate whether MVs can protect bacteria against β‐lactam antibiotics, antibiotic‐sensitive bacteria RC85 were cultured with MVs from RC 85‐T in an environment with above normal MIC for ampicillin. The growth kinetics showed that RC85 was able to grow when treated with RC85‐T MVs but did not grow when treated with RC85 MVs (Figure 6a). Growth kinetics clearly showed that RC85‐T MVs protected RC85 in a dose‐dependent manner, allowing the bacteria to tolerate antibiotic exposure above MIC levels (Figure 6b). To investigate whether MVs degraded or directly affected the antibiotics, LC‐ESI‐QQQ analysis was performed at 3, 6, and 12 h incubation of MVs in a cell‐free system to measure concentrations of ampicillin (Figure 7). The concentration of ampicillin in samples treated with RC85‐T MVs decreased over time, but the concentration of ampicillin in samples treated with RC85 MVs remained the same. These results indicate that the MVs clearly harbour β‐lactamase activity.

### Total protein obtained from *E. coli* RC85‐T and *E. coli* RC85 MVs

To find out which proteins are associated with β‐lactam resistance in RC85‐T MVs, the protein profiles of RC85‐T and RC85 MVs were compared by LC–MS/MS analysis. A total of 332 proteins were identified using the uni_bacteria database. RC85‐T MVs alone have 245 proteins and specifically, 184 proteins overlapped with RC85 MVs. The proteins analysed from the MVs of RC85‐T and RC85 cells consisted of various antimicrobial peptides as well as proteins related to antibiotic resistance (Table 3). Remarkably, two important proteins, OmpC and beta‐lactamase, which are involved in resistance against β‐lactam antibiotics were prominent only in RC85‐T MVs. Furthermore, antimicrobial‐resistant proteins such as OmpA, OmpT, MipA, LpoA, and LpoB were also only detected in RC85‐T MVs (Duval et al., 2009; Llobet et al., 2009; Stumpe et al., 1998; Xu et al., 2006; Zhang et al., 2008). Hence, the possible role of RC85‐T MVs in inhibiting the action of β‐lactam antibiotics in bacteria could be due to the presence of these relevant proteins.

**TABLE 3:** Quantitative protein profiling related to antibiotic resistance by comparing the membrane vesicles (MVs) secreted by RC85‐T and RC85 using LC‐ESI‐MS/MS

Identified proteins	Accession number	Alternate ID	Identification	
			RC85‐T MVs	RC85 MVs
1 Beta‐lactamase	A0A097SR23	Bla	1.2317	0
2 Outer membrane protein C	A0A069XT05 (+12)	OmpC	824.03	423.35
3 Outer membrane protein C	A0A0H3CMG2	OmpC	142.88	62.283
4 Outer membrane protein C	Q5ETV5	OmpC	310.4	191.9
5 Outer membrane protein A	A0A024L4C3 (+48)	OmpA	1331.50	1004.90
6 Outer membrane protein A	A0A0J8XTY7 (+3)	OmpA	1008.80	775.17
7 Outer membrane protein A	A0A061YKB8 (+7)	OmpA	1035.90	783.58
8 Outer membrane protein A	A0A0E2L894 (+6)	G925_00949	1112.20	857.65
9 Outer membrane protein A	A0A3U8E3T6	DMC44_11945	997.70	770.96
10 Outer membrane protein	A0A3A1QF28	C9Z70_14080	1225.60	931.71
11 Outer membrane protease	A0A423Y0J4	OmpT	532.11	0
12 Outer membrane protease	A0A0K4LE39	OmpT	466.82	0
13 MipA/OmpV family protein	A0A0K4LE39	MipA	52.96	0
14 MipA/OmpV family protein	A0A377CK31	MipA_1	56.66	0
15 Penicillin‐binding protein activator LpoA	A0A069FNZ6 (+52)	LpoA	140.42	66.491
16 Penicillin‐binding protein activator LpoB	A0A2X3KBR9	LpoB	4.93	0

## DISCUSSION

In the present study, we investigated whether MVs from Gram‐positive bacteria could transfer antibiotic‐resistant substances to antibiotic‐sensitive Gram‐negative bacteria. Compared to a first study showing β‐lactamase activity of MVs from *S. aureus* (Lee et al., 2013), this study further demonstrated that a β‐lactam‐resistant *E. coli* (RC85‐T) was produced from an antimicrobial‐sensitive *E. coli* (RC85) by MV‐mediated transfer of antibiotic resistance from a methicillin‐resistant *S. aureus*, MRSA ST541. We then compared MVs obtained from both RC85‐T and RC85 cells to see if the same strains of *E. coli* possess different traits, including antibiotic resistance. We isolated both MVs from RC85‐T and RC85 cells to examine their ability to degrade antibiotics. Our results showed that MVs could deliver substances involved in antibiotic resistance and that RC85‐T MVs could break down β‐lactam antibiotics leading to higher MICs than RC85 MVs.

Several studies have demonstrated that vesicles secreted by bacteria hydrolyse antibiotics or act as decoys for antibiotics, thereby protecting the bacteria against antibacterial peptides and antibiotics (Manning & Kuehn, 2011; Mashburn‐Warren & Whiteley, 2006; Schaar et al., 2011; Wagner et al., 2018). Some MVs possess β‐lactamase enzymes that can hydrolyse β‐lactam antibiotics, which give other bacteria the ability to survive in the presence of the antibiotic (Rumbo et al., 2011). Consistent with these studies, we demonstrated that MVs purified from MRSA ST541 and RC85‐T cells had the ability to protect the antibiotic‐susceptible bacteria to survive in the presence of ampicillin. Both growth curves and viable bacterial counts showed that MVs from RC85‐T cells were able to help β‐lactam‐sensitive RC85 cells grow in the presence of ampicillin in a dose‐dependent manner. The LC‐QQQ analysis of ampicillin in a cell‐free system in the presence of the MVs showed that MVs from RC85‐T cells, but not from RC85 cells, could degrade β‐lactam antibiotics over time. Analysis of the protein composition of MVs from both RC85‐T and RC85, by LC‐ESI‐MS/MS revealed differential expression of several proteins potentially involved in the breakdown of β‐lactam antibiotics. Particularly, both Bla and OmpC were upregulated in the RC85‐T MVs but not in RC85 MVs. Bla is a β‐lactamase that can inactivate β‐lactam antibiotics (Domingues & Nielsen, 2017; Kim et al., 2018; Lee et al., 2013; Schaar et al., 2011). The RC85‐T MVs showed antibiotic resistance by mediating the influx of β‐lactam antibiotics into MVs through increased OmpC (Devos et al., 2016; Wilke et al., 2005). Other antimicrobial‐resistant proteins such as OmpA, OmpT, MipA, LpoA, and LpoB, would contribute to inactivate some β‐lactam antibiotics by the β‐lactamase located in the lumen of MVs (Duval et al., 2009; Llobet et al., 2009; Park et al., 2016; Stumpe et al., 1998; Xu et al., 2006; Zhang et al., 2008). These proteins would explain how β‐lactam‐sensitive *E. coli* can persist even though they were treated with the lethal dose of β‐lactam antibiotic by the presence of RC85‐T MVs.

Bacteria can convey their contents to other bacteria through MVs (Fulsundar et al., 2014; Kadurugamuwa & Beveridge, 1999; Kim et al., 2018; Kim, Jang, et al., 2015; Kim, Lee, et al., 2015; Kulkarni et al., 2015; Lee et al., 2013; Meyer & Fives‐Taylor, 1993). Various roles have been identified from MVs in terms of protein delivery, horizontal gene transfer (HGT), cell‐to‐cell communication, and bacterial defence (Fulsundar et al., 2014; Liu et al., 2018; Yaron et al., 2000). Among them, studies have suggested that MVs can transfer specific genes to other bacteria via MVs‐mediated HGT process, including antibiotic‐resistant genes (Domingues & Nielsen, 2017; Lee et al., 2013; Liu et al., 2018; Soler & Forterre, 2020; Yaron et al., 2000). Using MIC profiling, we demonstrated that *E. coli* RC85‐T cells acquired resistance to some β‐lactam antibiotics after incubation with MVs from MRSA ST541 cells. Indeed, further LC‐QQQ‐based analysis revealed a significant increase in β‐lactamase levels, not only in RC85‐T cells but also in their MVs. We observed a 12‐times ratio in activity between MVs of RC85‐T compared to MVs of RC85, whereas this ratio was only 3.3 between RC85 and RC85‐T cells. This could correspond to a higher expression of the protein in RC85‐T cells and/or to a higher recruitment of beta‐lactamase during the production of MVs in RC85‐T compared to RC85 cells.

Our results demonstrated that substances involved in conferring β‐lactam antibiotic resistance were transferred to RC85 cells through MVs from MRSA ST541, although the substances involved remain to be identified. Our future studies will clarify what the substances in MVs are using the functional genomic approaches. In addition, the present study found that RC85‐T cells exhibited selective β‐lactam antibiotic resistances since the cells were resistant to ampicillin, but sensitive to the third generation cephalosporins such as cefoperazone and cefotaxime (Table 2). However, MRSA 541 cells were resistant to ampicillin, cefoperazone and cefotaxime (Table S1). These selective bacterial antibiotic resistances transferred by MVs also need to be clarified. Taken together, the present study indicates that MVs play a crucial role in bacterial survival against lethal doses of antibiotics, by not only inactivation of antibiotics through enzyme activity, but also transferring genetic substances to adjacent antibiotic‐sensitive bacteria (Table 3).

In conclusion, we showed that antibiotic‐resistant substances can be transferred to antibiotic‐susceptible bacteria through MVs. Vesicles are important mediators of antibiotic resistance, transmitting antibiotic resistance to susceptible bacteria and thus giving bacteria protection against β‐lactam antibiotics. More importantly, our study demonstrated the successful transfer of antibiotic resistance through MVs from MRSA ST541, a Gram‐positive bacterium, to the antibiotic‐susceptible *E. coli* RC85, a Gram‐negative bacterium. Our findings help shape our knowledge of the significance of vesicles in the emergence of resistant bacterial strains from a mixed bacterial community. Understanding the mechanism involved in bacteria developing resistance to antibiotics could help in the development of effective tools to control the spread of MDR bacteria.

## CONFLICT OF INTEREST

The authors declare no conflict of interest.

## Supporting information


**Table S1‐S2** Supporting information
