# Supplementary material for: Membrane vesicles from antibiotic‐resistant Staphylococcus aureus transfer antibiotic‐resistance to antibiotic‐susceptible Escherichia coli
Source: J Appl Microbiol. 2022 Feb 11;132(4):2746–59. doi: 10.1111/jam.15449 (PMC9306644; doi:10.1111/jam.15449)
Supplement: Supplementary file 1 — Table S1‐S2 Supporting information [file JAM-132-2746-s001.docx]

**Supporting information**

**Table S1. The MIC of several antibiotics against the β-lactam-susceptible *Staphylococcus aureus* ATCC29213 and β-lactam-resistant *S. aureus* ST541**

|  |  |  |  |
| --- | --- | --- | --- |
| Class | Antibiotics | MIC (μg/mL) | |
|  |  | ST541 | ATCC29213 |
| β-Lactams | Ampicillin | 32 | 2 |
|  | Cefoperazone | 16 | 4 |
|  | Cefotaxime | 4 | 1 |
| Aminoglycosides | Gentamicin | >256 | 4 |
|  | Kanamycin | >256 | 16 |
|  | Streptomycin | >256 | 32 |
| Polymyxins | Colistin | >256 | >256 |
| Tetracyclines | Tetracycline | 128 | 1/2 |

**Table S2. Identification of colonies by using MALDI-Biotyper. RC85-T cell acquired antibiotic resistance after exposed to MVs of the indicated MRSA ST541.**

|  |  |  |  |
| --- | --- | --- | --- |
| No. | Strain | Matching pattern | Score value |
| 1 | RC85-T | *Escherichia coli* | 1.983 |
| 2 | RC85-T | *Escherichia coli* | 2.195 |
| 3 | RC85-T | *Escherichia coli* | 2.119 |
| 4 | RC85-T | *Escherichia coli* | 2.267 |
| 5 | RC85-T | *Escherichia coli* | 2.185 |
| 6 | RC85-T | *Escherichia coli* | 2.218 |
| 7 | RC85-T | *Escherichia coli* | 2.346 |
| 8 | RC85-T | *Escherichia coli* | 2.293 |
| 9 | RC85-T | *Escherichia coli* | 2.235 |
| 10 | RC85-T | *Escherichia coli* | 2.234 |
|  |  |  |  |

The MALDI-Biotyper 3.0 software, that was used to process the raw spectra, assigns a log10 score of 0 to 3.0 according to spectral peak patterns: 0 to 1.699 is interpreted as no reliable identification; 1.700 to 1.999 indicates a probable genus-level identification; 2.000 to more indicates a secure genus-level identification and a probable or highly probable species-level identification.
